# DERMATOSES IN THE EARLY NEONATAL PERIOD: THEIR ASSOCIATION WITH NEONATAL, OBSTETRIC AND DEMOGRAPHIC VARIABLES

**DOI:** 10.1590/1984-0462/;2019;37;3;00012

**Published:** 2019-06-03

**Authors:** Elisa Maria Michels Krüger, Fernanda Sinkos, Julia Feldmann Uhry, Julio Cesar Bezerra De Boni, Cristina Terumi Okamoto, Kátia Sheylla Malta Purin, Renato Nisihara

**Affiliations:** a Universidade Positivo, Curitiba, PR, Brazil.

**Keywords:** Dermatosis, Pediatrics, Infant, newborn, Dermatoses, Pediatria, Recém-nascido

## Abstract

**Objective:**

To evaluate the prevalence of neonatal dermatoses in the early neonatal period and to associate them with neonatal, demographic and obstetric variables.

**Methods:**

A cross-sectional study with neonates and their respective mothers, who were hospitalized in a public maternity hospital in Curitiba, PR, Brazil. Data collection was performed using information present in the medical records and a physical examination of the newborn during the period between April 2015 and May 2016.

**Results:**

350 neonates were evaluated. 54.8% were male, and 94.8% (332/350) presented a dermatosis. Among them, 84.6% had, concomitantly, two or more dermatoses. A total of 23 types of dermatoses were diagnosed. The most prevalent were: sebaceous hyperplasia (66%); fluff (42.6%); and salmon patches (41.4%). The mean age of the mothers was 24.9±4.9 years old, and they were predominately white (57.7%). Vernix caseosa was associated with the female gender (p=0.034). Nonwhite mothers were associated with genital hyperpigmentation (p=0.03) and Mongolian spots (p=0.001). Physiological flaking was associated with cesarean deliveries (p=0.03) and a gestational age of over 40 weeks (p=0.054). Salmon patches was associated with primiparity (p=0.0001).

**Conclusions:**

There was a high prevalence of neonatal dermatosis in the studied population. Each newborn had, on average, three different dermatoses. Dermatosis in neonates was associated with primiparity, nonwhites, a gestational age of over 40 weeks, and the sex of the newborn.

## RESUMO

**Objetivo:**

Avaliar a prevalência de dermatoses no período neonatal precoce e associar sua ocorrência a variáveis neonatais, demográficas e obstétricas.

**Métodos:**

Estudo transversal com neonatos e respectivas puérperas internados em alojamento conjunto de uma maternidade pública de Curitiba (PR). A coleta de dados foi realizada por meio de informações presentes nos prontuários e no exame físico do recém-nato durante o período de abril de 2015 a maio de 2016.

**Resultados:**

Foram avaliados 350 recém-nascidos, 54,8% do sexo masculino, e 332 (94,8%) apresentaram dermatoses. Desses 332, 84,6% tiveram, concomitantemente, duas ou mais dermatoses. Diagnosticou-se o total de 23 tipos de dermatoses, sendo mais prevalentes: hiperplasia sebácea (66,6%); lanugem (42,6%); e mancha salmão (41,4%). A média de idade das puérperas foi de 24,9±4,9 anos, com predomínio de etnia branca (57,7%). Vérnix foi associado a sexo feminino (p=0,034). A etnia materna não branca associou-se à hiperpigmentação genital (p=0,030) e mancha mongólica (p=0,001). A descamação fisiológica associou-se ao parto cesáreo (p=0,030) e à idade gestacional acima de 40 semanas (p=0,054); e mancha salmão, à primiparidade (p=0,0001).

**Conclusões:**

Verificou-se alta prevalência de dermatose neonatal na população estudada, sendo cada recém-nascido acometido em média por três tipos. Houve associação da presença de dermatoses com primiparidade, mães não brancas, idade gestacional superior a 40 semanas e sexo do neonato.

## INTRODUCTION

The early neonatal period is marked by the newborn adapting to extrauterine life, with physiological changes that result because of changes involved with moving from the liquid uterine environment to the external dry environment. During this period, dermatological conditions may be present due to the peculiarities of neonatal skin. Its cutaneous constitution differs from adult skin because of its glandular and melanocytic immaturity, its thin layer thickness, and its biochemical composition, which alkalinizes the pH. These anatomical and physiological factors make it predisposed to neonatal dermatoses (ND). Because of the benign and self-limiting physiological nature of most lesions, there is no need for any intervention. Avoiding excessive handling of the newborn is recommended because of the potential risk for iatrogenesis.^1^

The appropriate characterization of the highly prevalent NDs in the newborn population treated at our hospitals improves the differential diagnosis of pathogenic eruptions of vertical transmission that require intervention.^2^ In addition, the correct diagnosis reduces the need for dermatological outpatient clinics, where it is estimated that 30% of consultations deal with this condition.^3^ Recognizing NDs is essential to reassure parents about physiological skin changes and avoid unnecessary therapeutic measures.^4^

The present study had as its objectives to evaluate the prevalence of NDs in the early neonatal period and to verify associations between the presence of such changes with factors related to gestation and characteristics of the newborn (NB).

## METHOD

The study was approved by the Committee of Ethics in Research (CEP) of the *Hospital do Trabalhador* and the Health Secretariat of Paraná (SES / PR), and was registered under the Certificate of Presentation for Ethical Assessment (CAAE) number 44236115.8.0000.5225.

The maternity hospital, *Hospital do Trabalhador,* was selected as a reference, given its high birth rate/year. Since the implantation of the Mãe Curitibana Program in 1999, it is a reference for 26 health units in the state capital.

The sample size was calculated based on the average of three thousand births per year in the hospital, totaling 350 newborns and 350 new mothers, and giving a sample power of over 95%. It was a cross-sectional study carried out between April 2015 and May 2016, through the clinical evaluation of dermatological alterations in a consecutive sample of NBs up to seven days old. In addition, data were collected from the records of the newborn and the new mother with regard to birth conditions and gestational data. Both the newborn and the new mother were hospitalized in the maternity unit.

The criteria for inclusion were the following: NB of up to seven days of life and their respective mother; they agreed to and signed the free and informed consent form along with the person in charge. The exclusion criteria were: patients with incomplete data in their medical records; mucosal dermatoses and faneros; neoplastic and infectious dermatoses; postpartum women under 18 years of age; and patients admitted to the Intensive Care Unit (ICU).

All of the researchers were trained by an experienced dermatologist to inspect the skin of the NB, and were able to perform a dermatological diagnosis of the ND. In cases of doubt, photographs of the dermatosis were taken for further discussion and decision making. The diagnosis was exclusively clinical, and did not involve biopsy procedures. The diagnosed skin changes were grouped in the following categories, based on criteria of pediatric dermatology described by Hulsmann et al.^5^ and Kane et al.^6^:

- General: vernix caseosa, fluff, sebaceous hyperplasia, miliaria crystalline, physiological flaking, neonatal cephalic pustulosis, neonatal toxic erythema.
- Local: diaper erythema, seborrheic dermatitis.
- Vascular: salmon patches, telangiectasia, marmoreal cutis, generalized erythema, plethora, acrocyanosis, childhood hemangioma.
- Pigmentation: Mongolian spots, hyperpigmentation of the genitalia, melanocytic nevus, café au lait spots.

The data obtained were arranged in Microsoft Excel spreadsheets, and the results were expressed as mean and standard deviation. The dichotomous variables were analyzed by the chi-square test and Fisher’s exact test using the GraphPad Prism 5.0 statistical package. Values less than 5% were considered to be significant.

## RESULTS

In total, 362 cases were evaluated. For 12 of them, the medical records were incomplete, and were excluded. Thus, a total of 350 new mothers and neonates were included. When evaluating data from the new mothers, the age range of 22-30 years old prevailed (53.1%), with the mean age of the women studied being 24.9±4.9 years old. Among the women evaluated, 202/350 (57.7%) declared themselves to be white, 125/350 (35.7%) declared themselves to be brown and 23/350 (6.6%) declared themselves to be black. With regard to the type of delivery, 260/350 (74.3%) had a vaginal delivery and 90/350 (25.7%) had a cesarean delivery. In the sample, 217/350 (62%) were multiparous and 133/350 (38%) were primiparous.

Among the 350 newborns examined, 192 (54.8%) were males and 236 (67.4%) were between zero and one day old (mean 1.24 days). With regard to weight, 315 (90%) were between 2,500 and 3,999 g, with a mean of 3,245.4±442.57 g. Gestational age at the time of delivery for 98% of the sample was between 37 and 41 weeks.

**Figure 1:**
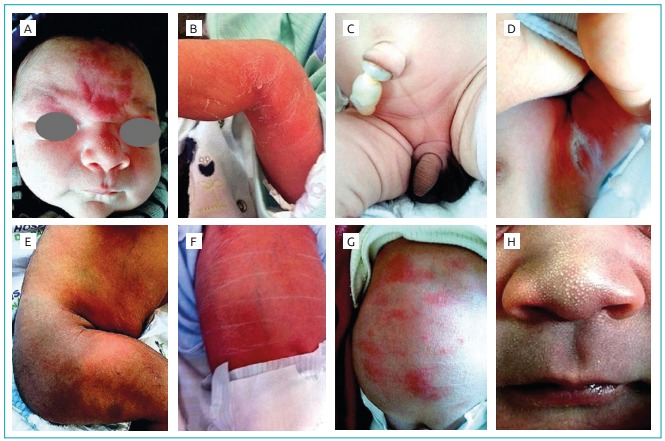
Neonatal dermatoses observed in the studied group.

The presence of ND was found in 94.8% of the evaluated NBs, and in 295 (84.6%) there was more than one type of cutaneous development. Twenty-five different types of ND were found, and their diagnostic frequency was 1,028. Figure 1 illustrates some of the ND found. In relation to the categories, the prevalence of the following were: general (71.4%), vascular (19%), pigment (7%), local (1.9%), malformation (0.19%) and tocotraumatism (0.28%). This amount was obtained by adding up the total number of dermatoses verified in the 332 affected NB, resulting in an average of three concomitant neonatal dermatosis for each patient. No NB of the study needed additional treatment because of their dermatosis. The types of ND observed in the present study are described in Table 1.

**Table 1:** Distribution of neonatal dermatoses according to the categories identified in the 350 newborns.

Dermatosis by category	n	Frequency (%)
General		
Sebaceous Hyperplasia	233	66.6
Physiological flaking	140	40
Fluff	149	42.6
Vernix caseosa	91	26
Crystalline miliaria	89	25.4
Toxic erythema	18	5.1
Neonatal cephalic pustulosis	7	2
Local		
Diaper erythmea	19	5.4
Seborrheic dermatitis	1	0.3
Vascular		
Salmon patches	145	41.4
Telangiectasia	18	5.1
Cutis marmorata	9	2.6
Generalized erythema	6	1.7
Childhood hemangioma	3	0.8
Pigment		
Genital hyperpigmentation	39	11.1
Mongolian spots	32	9.1
Melanocytic nevus	2	0.8
Café au leite spots	1	0.3
Total	1,028	

The prevalence of ND varied according to the sex of the newborn. Genital hyperpigmentation, one of the representatives of the pigment category, was significantly more present in males (p<0.001). On the other hand, vernix caseosa was significantly more observed in females (p=0.033). For the other dermatoses, there was no significant difference between the sexes.

The delivery method and parity influenced the frequency of ND. There was a higher frequency of ND in the general, vascular and pigment categories in recent mothers with only one child. With regard to the delivery method, in the general and local categories, there was a higher prevalence of neonatal dermatoses in vaginal and cesarean birth infants, respectively. Data are shown in Table 2.

**Table 2:** Distribution of neonatal dermatoses by category according to parity and delivery method.

Dermatosis	Parity			%	p-value	Delivery method			%	p-value
	Multiparous n=217	%	Primiparous n=133			Vaginal n=260	%	Cesarean n=90		
General										
Sebaceous hyperplasia	144	66.3	89	66.9	1	179	68.8	54	60	0.92
Physiological flaking	88	40.5	52	39	0.91	104	40	36	40	1
Fluff	84	38.7	65	48.8	0.27	109	41.9	31	34.4	0.42
Vernix caseosa	51	23.5	40	30	0.33	73	28	18	20	0.27
Crystalline miliaria	52	23.9	37	27.8	0.54	67	25.7	22	24.4	0.89
Toxic erythema	13	5.9	5	3.75	0.46	11	4.2	7	7.7	0.27
Local										
Diaper erythema	8	3.6	11	8.2	0.09	12	4.6	7	7.7	0.29
Seborrheic dermatitis	1	0.4	0	0	1	0	0	1	1.1	0.25
Vascular										
Salmon patches	62	28.5	83	62.4	<0.001	107	41.1	38	42.2	0.91
Cutis marmorata	4	1.8	5	3.7	0.31	7	2.6	2	2.2	1
Generalized erythema	1	0.4	5	3.7	0.034	4	1.5	2	2.2	0
Pigment										
Genital hyperpigmentation	22	10.1	17	12.7	0.60	33	12.6	6	6.6	0.17
Mongolian spots	13	5.9	19	14.2	0.023	26	10	6	6.6	0.40

The dermatoses had different sites of development. Salmon patches affected 30% of cases and more than one anatomical region, with 62.8% in the eyelid and 28.3% in the glabellar region. Sebaceous hyperplasia was limited to the face, with 86.3% of cases in the nasal region. Table 3 shows the skin changes and the body sites where they were observed.

**Table 3:** Distribution of dermatoses according to anatomical regions.

Dermatosis	Total	Front n=492	%	Back n=135	%	LL and LLF n=232	%	UL and ULF n=92	%	Torso n=51	%	Genitals n=8	%	LBS n=29	%
Sebaceous Hyperplasia	233	233	47.3	0	0	0	0	0	0	0	0	0	0	0	0
Physiological flaking	194	23	4.6	4	2.9	97	41.8	29	31.5	3	5.8	0	0	0	0
Fluff	196	11	2.2	108	80	45	19.3	42	45.6	1	1.9	0	0	0	0
Vernix caseous	106	10	2	1	0.7	44	18.9	10	10.8	30	58.8	8	100	0	0
Crystalline miliaria	120	53	10.7	7	5.1	20	8.6	1	1	9	17.6	0	0	0	0
Toxic erythema	35	11	2.2	4	2.9	10	4.3	1	1	2	3.9	0	0	0	0
Salmon patches	145	145	29.4	0	0	0	0	0	0	0	0	0	0	0	0
Cutis marmorata	9	0	0	1	0.7	6	2.5	2	2.1	0	0	0	0	0	0
Generalized erythema	36	6	1.2	6	4.4	6	2.5	6	6.5	6	11.7	0	0	0	0
Mongolian spots	38	0	0	4	2.9	4	1.7	1	1	0	0	0	0	29	100

LL: lower limb; LLF: lower limb fold; UL: upper limb; ULF: upper limb fold; LBS: lumbosacral.

Through dichotomous statistical analysis, each neonatal, demographic and obstetric variable, listed in Tables 1 and 2, was assigned to each of the 23 neonatal dermatoses identified in the series. Table 4 shows the significant associations, demonstrating each variable that constituted a risk factor for the appearance of the specific neonatal dermatosis listed.

**Table 4:** Significant associations between the dermatoses and obstetrical, puerperial and neonatal variables.

Dermatosis	Obstetric profile	OR (p-value)	Puerperal profile	OR (p-value)	Neonatal profile	OR (p-value)	Dermatologic profile	OR (p-value)
Salmon patches	Primiparous	21.8 (p<0.001)						
Physiological flaking	Cesarean birth	16,7 (p=0.03)			IG>40 weeks	0.6 (p=0.054)		
Genital hyperpigmentation			Non-white	2,1 (p=0.03)	Masculine	7.2 (p<0.001)		
Mongolian spots			Non-white	3,9 (p<0.001)				
Vernix caseous					Feminine	1,0 (p=0.034)	No flaking	1.9 (p<0.001)

OR: Odds Ratio; GI: gestational age.

## DISCUSSION

The high prevalence of ND and their multiple and concomitant form of development match what is said in the medical literature. In addition, they are intrinsically related to the ethnic, demographic and obstetric profiles of the populations studied. It is extremely important that, in addition to dermatologists, professionals directly involved in the primary care of newborns are able to diagnose these physiological changes. DN may occur because the cutaneous attachments that are still immature at birth gradually pass through a maturation process in the early neonatal period. As such, the skin plays a role in adapting to extrauterine life.^4^ ^,^ ^7^

At the national level, there are investigations with the neonatal population of Rio Grande do Sul, Rio de Janeiro, São Paulo and Pernambuco.^7^ ^,^ ^8^ ^,^ ^9^ ^,^ ^10^ The high frequency of ND observed in the present study (94.8%) was similar to the other Brazilian studies. The rate of multiple cutaneous development (84.6%) was also high, similar to the other studies in which it ranged from 48.1 to 87.9%.^7^ ^,^ ^9^ ^,^ ^10^ Therefore, it is common for newborns to have ND, mostly multiple and concomitant types.

Internationally, there are many studies on the subject. The prevalence of ND ranged from 34.7%^14^ in a Pakistani study to 99.3% in Australia.^15^ Intermediate values were observed in India (55.0%)^3^ and Turkey (67.3%).^16^ The values that most approached our prevalence were those recorded in the Iranian (96%)^11^ and Indian populations (93.3%).^12^ The high prevalence demonstrates that skin adaptation is an intrinsic phenomenon to human development, even despite ethnic differences and cutaneous peculiarities.^11^ ^,^ ^17^ ^,^ ^18^ ^,^ ^19^ Differences between ND data in different populations may occur due to methodological and ethnic-social variations, such as: sample size, term of the neonatal period when performing the examination, inclusion of pathological dermatoses, inclusion of mucosas/faneros and ethnic plurality.^12^ Sanitary infrastructure conditions are also highlighted, since in Indian regions, the prevalence of infectious parasitic dermatoses overlaps with physiological dermatoses.^3^

In quantitative terms, 23 types of dermatoses were diagnosed in the present study, a value similar to that described in the literature, which varied from 15 to 35.^8^ ^,^ ^10^ ^,^ ^12^ The prevalence of the six dermatological categories were, in descending order: general (71.4%), vascular (19%), pigment (7%), local (1.9%), malformations (0.19%) and tocotraumatism (0.28%). The first three categories mentioned made up 97.4% of the 23 manifestations, so they should be a focus of attention for newborn skin. This pattern was repeated in an Indian study,^18^ in which general dermatoses were among the 10 most prevalent diagnoses. Dermatoses of the general and pigment categories were the most prevalent^9^ ^,^ ^10^ ^,^ ^19^ ^,^ ^20^ in national and international studies. Our study corroborates these findings, since, of the five predominant types of ND, four belonged to the general category. There is no consensus in the literature regarding the most common dermatosis in the general category, since in each study analyzed, one of the following DNs was cited as most prevalent: flaking,^19^ toxic erythema,^3^ hyperplasia sebaceous^9^ ^,^ ^17^ ^,^ ^21^ and fluff.^8^ ^,^ ^9^ There was agreement between our study and others when comparing the vascular and pigment categories, taking into account that the salmon patches^9^ ^,^ ^19^ ^,^ ^21^ and Mongolian spots^9^ ^,^ ^14^ ^,^ ^20^ ^,^ ^21^ were, respectively, the most common. There was disagreement with regard to the pigmentary dermatoses: in our study, genital hyperpigmentation prevailed, and in the others, Mongolian spots was the most represented in the category.^9^ ^.^ ^14^ ^.^ ^20^

Sebaceous hyperplasia had a similar rate as in the literature^11^ ^,^ ^14^ ^,^ ^19^ ^,^ ^21^ and was the most prevalent in the study. The development and function of the sebaceous gland in the fetus and neonate are regulated by maternal androgen and the synthesis of endogenous steroids, which lead to increased sebaceous excretion within a few hours after birth. This fact can cause excessive proliferation and superficial visualization at the beginning of extrauterine life.^22^ There was no statistical significance between sebaceous hyperplasia and maternal gestational intercurrences, which differs from other studies.^11^ Although reported as risk factors, in our study, multiparity^20^ and males were not predisposed to dermatosis.

Salmon patches (a type of vascular capillary malformation) has a pinkish-reddish tint, accentuates crying, and disappears after digital pressure. As found in 30% of the sample, it commonly affects more than one site,^23^ concentrating in the eyelid and glabellar region, which is similar to the findings of Indian research.^17^ Salmon patches is considered the main vascular dermatosis, and the reported frequency is between 20 and 28%.^8^ ^,^ ^12^ ^,^ ^17^ ^,^ ^21^ ^,^ ^23^ ^,^ ^24^ In our sample, such DN was present in 41.4% of NB, which higher than that observed in other populations. Some authors have described multiparity and being male as risk factors for ND. However, this was not observed in our study. On the other hand, primiparity made newborns predisposed to ND, doubling its chance of appearance. Differing from other authors,^8^ ^,^ ^25^ there was no association between maternal age above 35 years old and the female sex.

Mongolian spots are a hyperpigmentation of blue-gray tones that arise from the migratory flow of melanocytes during the embryonic stage.^5^ ^,^ ^6^ ^,^ ^26^ ^,^ ^27^ The wide range of this DN’s prevalence (between 20 and 89%) reflects the ethnic differences between countries, since it has a higher frequency in populations that have skin with a higher phototype (from 72 to 89%).^3^ ^,^ ^17^ ^,^ ^19^ The Mongolian spots in the present study had a higher incidence in males in the lumbosacral region, and were associated with neonates of non-white new mothers (p=0.0005; *Odds Ratio* - OR=3.9), which is in agreement with other studies.^8^ ^,^ ^17^ ^,^ ^16^ ^,^ ^26^ ^,^ ^27^ It is worth noting the high rate of black and brown women (42.3%) in our sample. This is different from the census figures that come from the Brazilian Institute of Geography and Statistics (*Instituto Brasileiro de Geografia e Estatística* - IBGE) for the capital city of the state of Paraná, in which the percentage is 23%.^28^ Seventy-seven percent of the population of Curitiba and the surrounding region is white.^28^ One explanation for this finding may be the great migratory flow of Haitians to the capital of Paraná, which has occurred in the last decade. Currently, according to IBGE data, the number of Haitians living in Curitiba and the metropolitan region is estimated at 4,000 people.^29^ Haitian pregnant women are referred to the *Hospital do Trabalhador* because they have available beds and specific triage procedures for early care obstetrics. It is a reference hospital for 26 health units in the Paraná State capital.^29^

Transient hyperpigmentation of genitalia occurs when the melanocyte stimulating hormone is exacerbated during intrauterine life, increasing the number of dendritic cells containing melanin granules and reducing the action of melanophages. The prevalence observed in the present study was similar to that of the Gaucho study (18.9%).^6^ Furthermore, the Mongolian spots prevailed in NB of nonwhite recent mothers, and was associated with males, which corroborates data from the literature.^6^ ^,^ ^13^ ^,^ ^14^ ^,^ ^20^

Diaper dermatitis occurs when the perineal epidermal barrier is changed. It is influenced by cutaneous care such as: local occlusion, fecal enzymes, topical products and friction during local hygiene.^24^ ^,^ ^30^ Its prevalence in our study was similar to that in others.^11^ ^,^ ^18^ ^,^ ^20^ ^,^ ^30^ Taking into account that more than half of the newborns wearing diapers had at least one episode until their weaning,^30^ we considered the prevalence of 5.4% to be high since the average days of life (1.24) and the period of analysis (seven days) were short.

The prevalence of physiological flaking described in the literature ranges from 1.9 to 83%. In our sample, the diagnosis was made in 40% of the NBs, coinciding with other investigations.^11^ ^,^ ^12^ ^,^ ^17^ We observed an association with delivery method, since having a cesarean section and a gestational age over 40 weeks increased the chance of occurrence. There was no association with neonatal sex and parity.

Vernix caseosa was diagnosed in 26% of the studied NB, and the frequency described by other authors ranged from 7.7 to 49.2%.^17^ ^,^ ^31^ It has been shown, as well as in other investigations^31^ that the absence of vernix caseosa indicated a predisposition for flaking (p<0.001; OR=1.9). Biochemical analyzes detected a greater amount of amino acids in areas of skin with vernix, conferring more hydration of the skin of the NB and preventing flaking.^32^ In addition, a higher prevalence was observed in females (p=0.034; OR=1.7). Vernix is an indication of fetal maturity and epidermal synthesis because the levels of its production, along with that of ceramides, rise between 39 and 40 weeks.^33^ ^,^ ^34^ ^,^ ^35^ Although described,^17^ the association of vernix and gestational age over 40 weeks was not observed in our study. This is because, from this intrauterine stage, the vernix can detach itself in the amniotic fluid, before birth.

The neonatal toxic erythema is characterized as a sterile erythematous halo. They are single or multiple papules that are predominate on the face, torso and extremities.^2^ ^,^ ^5^ ^,^ ^36^ Despite its low prevalence, it is important to identify it, because it is one of the differential diagnoses of candidiasis, genital herpes and syphilis. Some studies point to a frequency between 1.3 and 54%,^8^ ^,^ ^11^ ^,^ ^12^ ^,^ ^13^ ^,^ ^21^ ^,^ ^36^ and in our study the rate was 5.1%. Contrary to other studies, there was no association of neonatal toxic erythema with sex, type of delivery or parity.^8^ ^,^ ^12^ ^,^ ^16^ ^,^ ^17^

In the present investigation, there was a high prevalence of fluff (42.6%), higher than that described in the literature (7-25.7%). It is probable that this fact is due to the composition of the sample, with 98% of the newborns being full-term, considering that in this group, there is a predominance of DN.^12^ However, there is controversy regarding this topic.^8^ ^,^ ^12^ Although the male gender and adequate birth weight have been associated together,^12^ ^,^ ^20^ the present study did not find this association.

The limitations of the study are specific to cross-sectional research. Only a follow-up with the NB could confirm that all of dermatoses would in fact disappear within a few days. The dermatoses could have been confused with one another, but in these cases, the researchers relied on the opinion of an experienced dermatologist to confirm them.

This study also highlights the need for pediatric assistants, those who care first for the NB, to have significant knowledge of the physiological skin changes that are most prevalent in this population, so they can reassure the parents, and convey confidence that the baby has a dermatosis that will disappear on its own and will not require a specialist or the use of medication.

Finally, in this investigation, there was a high prevalence of ND - each NB was affected by three neonatal dermatoses on average. Regarding the variables inherent to gestation, there was a significant association between primiparity and salmon patches. In addition, there was one between nonwhite mothers and their child having genital hyperpigmentation and Mongolian spots. With regard to the newborns, a significant association was found between gestational age greater than 40 weeks and physiological flaking; genital hyperpigmentation and male sex; and vernix caseosa and the female sex.
